# 3D-printed NiFe-layered double hydroxide pyramid electrodes for enhanced electrocatalytic oxygen evolution reaction

**DOI:** 10.1038/s41598-021-04347-9

**Published:** 2022-01-10

**Authors:** Jinhyuck Ahn, Yoo Sei Park, Sanghyeon Lee, Juchan Yang, Jaeyeon Pyo, Jooyoung Lee, Geul Han Kim, Sung Mook Choi, Seung Kwon Seol

**Affiliations:** 1 Smart 3D Printing Research Team, Korea Electrotechnology Research Institute (KERI), Changwon-si, 51543 Gyeongsangnam-do Republic of Korea; 2 Electro-functional Materials Engineering, University of Science and Technology (UST), Changwon-si, 51543 Gyeongsangnam-do Republic of Korea; 3 Department of Energy and Electronic Materials, Surface Materials Division, Korea Institute of Materials Science, Changwon-si, 642831 Gyeongsangnam-do Republic of Korea; 4 Department of Materials Science and Engineering, Pusan National University, Busan, 46241 Republic of Korea; 5 KIURI Institute, Yonsei University, Seoul, 03722 Republic of Korea

**Keywords:** Energy science and technology, Engineering, Materials science

## Abstract

Electrochemical water splitting has been considered one of the most promising methods of hydrogen production, which does not cause environmental pollution or greenhouse gas emissions. Oxygen evolution reaction (OER) is a significant step for highly efficient water splitting because OER involves the four electron transfer, overcoming the associated energy barrier that demands a potential greater than that required by hydrogen evolution reaction. Therefore, an OER electrocatalyst with large surface area and high conductivity is needed to increase the OER activity. In this work, we demonstrated an effective strategy to produce a highly active three-dimensional (3D)-printed NiFe-layered double hydroxide (LDH) pyramid electrode for OER using a three-step method, which involves direct-ink-writing of a graphene pyramid array and electrodeposition of a copper conducive layer and NiFe-LDH electrocatalyst layer on printed pyramids. The 3D pyramid structures with NiFe-LDH electrocatalyst layers increased the surface area and the active sites of the electrode and improved the OER activity. The overpotential (*η*) and exchange current density (*i*_*0*_) of the NiFe-LDH pyramid electrode were further improved compared to that of the NiFe-LDH deposited Cu (NiFe-LDH/Cu) foil electrode with the same base area*.* The 3D-printed NiFe-LDH electrode also exhibited excellent durability without potential decay for 60 h. Our 3D printing strategy provides an effective approach for the fabrication of highly active, stable, and low-cost OER electrocatalyst electrodes.

## Introduction

Hydrogen is a promising and ideal fuel to replace fossil fuels because of its high energy density and pollution-free energy conversion characteristics. Although several methods have been developed for efficient hydrogen production, hydrogen generation from the separation of water into oxygen and hydrogen by electrochemical water splitting has been considered one of the most promising methods for clean and renewable energy production with no environmental pollution effects or greenhouse gas emissions^1–4^. Water splitting includes two half reactions, oxygen evolution reaction (OER, 4OH^−^→ O_2_ + 2H_2_O + 4e^−^) and hydrogen evolution reaction (HER, 2H_2_O + 2e^−^→ 2OH^−^ + H_2_) in alkaline media, which occur simultaneously at the anode and the cathode^5–7^. The standard reduction potential of the HER is defined as 0 V related to a relative hydrogen electrode (RHE), and the standard oxidation potential of the OER is 1.23 V (vs. RHE) at 25 °C and 1 atm^8^. Some factors related to the materials and devices (e.g., electrode morphology and conductivity, electrolyte diffusion blockage, bubble formation, and release) result in an additional potential over the standard one, which is called the overpotential (*η*).

Determining suitable processing parameters that can greatly decrease the *η* value and consequently promote the reaction rate and total cell efficiency is crucial in the water splitting process^9,10^. In previous reports, nanostructured and porous electrocatalysts with larger surface areas increased the number of active sites and contributed to alleviating the *η* value caused by the resistance of ions, gas diffusion, and charge transfer^11,12^. An effective approach for improving the performance of water electrolysis is to increase the total electrocatalyst–electrolyte interfacial active area by using a three-dimensional (3D) geometrical electrode coated with rational nanostructured electrocatalysts^13–16^. Although most current studies focus on the development of electrocatalyst materials, not the electrode form, diverse applications in the future will require a highly active, stable, and low-cost three-dimensional (3D) electrocatalyst electrode for water splitting.

Three-dimensional (3D) printing, also commonly referred to as additive manufacturing, is considering as a promising fabrication method since it enables rapid prototyping as well as the fabrication of free form-factor components^17–21^. Recently, several research groups have reported fabrication of effective 3D electrodes for water splitting applications using conventional 3D printing approaches such as a selective laser sintering (SLS) or a fused filament fabrication (FFF)^22–26^. However, it is difficult to reduce the sizes of the printed structures to a microscale in SLS and FFF approaches. On the other hands, an ink-based 3D printing approach enables to print 3D microstructures by a precise extrusion of specialized ink through a micro-nozzle^27–29^. It is possible to manipulate the surface area of the electrodes by producing 3D microstructures on the flat electrode surface.

Although the HER at the cathode is an important reaction for hydrogen generation by water splitting, the OER at the anode also plays a critical role in determining the steps for highly efficient water splitting because of the slow four-electron transfer kinetics of the anodic oxidation reaction^30^. Therefore, highly active anodes should be developed to produce hydrogen energy with high efficiency.

Here, we developed a new 3D printing strategy to produce a highly active 3D pyramid electrode for OER. The strategy consists of (i) an ink-based 3D printing approach using functional graphene ink and (ii) successive electrodeposition of the Cu conductive layer and NiFe-layered double hydroxide (LDH) electrocatalyst layer on a printed graphene pyramid array. The designed graphene ink composed of graphene and polymer solution was stable and suitable for an ink-extrusion 3D printing approach at a specific applied pressure (*P*). Compared to the flat electrodes with the same base area of 0.6 × 0.6 cm^2^, the 3D-printed pyramid electrode with a nanoparticle-based NiFe-LDH electrocatalyst had a relatively larger surface area, contributing to the increased number of active sites on the electrode. As a result, *η* and exchange current density (*i*_*0*_) of the NiFe-LDH pyramid electrode were 258 mV at 10 mA/cm^2^ and 0.818 μA/cm^2^, which were improved compared to those of the NiFe-LDH/Cu foil electrode. The 3D-printed electrode also showed excellent durability without potential decay for 60 h.

## Results

### Fabrication of the 3D-printed pyramid electrode for OER

**Figure 1:**
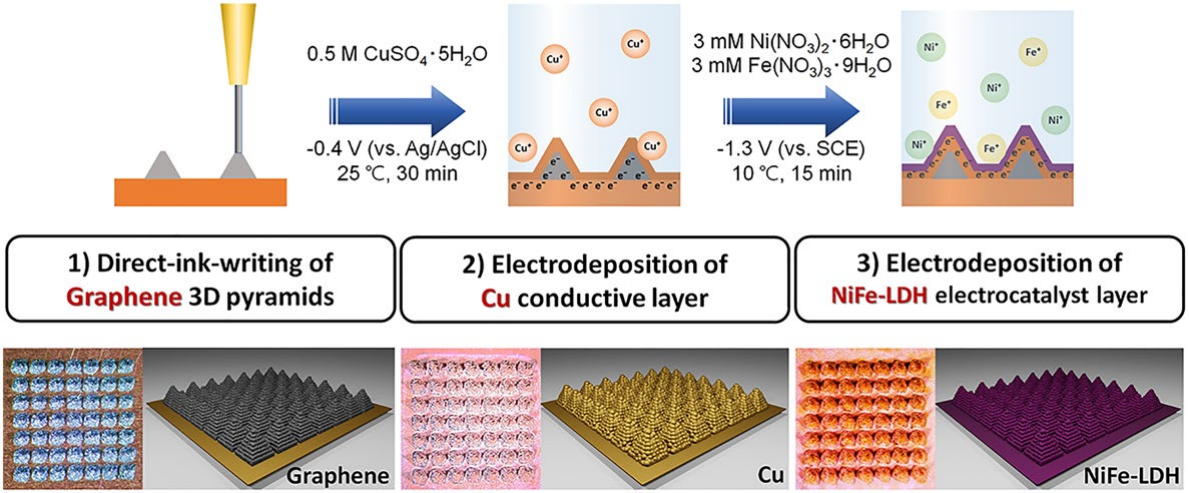
Schematic illustration of the fabrication of 3D-printed pyramid electrodes for OER in three successive steps. (1) Direct-ink-writing of graphene 3D pyramid array, electrodeposition of (2) Cu conductive layer and (3) NiFe-LDH electrocatalyst layers.

Figure 1 illustrates the fabrication process of the 3D-printed pyramid electrode with a NiFe-LDH electrocatalyst for OER. The electrode, comprising pyramid arrays, was prepared via a sequential process including ink-based 3D printing and electrodeposition, i.e., (i) direct-ink-writing of graphene 3D pyramid array and electrodeposition of (ii) Cu (conductive layer) and (iii) NiFe-LDH (electrocatalyst layer) on the printed pyramid array. Graphene 3D pyramids were printed onto a flat square Cu foil (base area = 0.6 × 0.6 cm^2^) with successive movements of the nozzle (inner diameter, *ID* = 100 μm) filled with a properly designed graphene ink (30 wt. % graphene microflakes, GMFs, and the 70 wt. % polymer solution). Each pyramid had a bottom area of 500 × 500 μm^2^, height (*h*) of 600 μm, and inclination angle of 67.4° (Movie S1). To evaluate the adhesion of the printed graphene to Cu foil, a simple tape test was conducted. The printed graphene pattern provides good adhesion to Cu foil (Fig. S1). This design enables a surface area twice larger than a flat electrode, thereby increasing the number of active sites on the electrode. For efficient deposition of the NiFe-LDH electrocatalyst on 3D electrodes, the Cu conductive layer was formed on the printed graphene pyramids through electrodeposition conducted at an applied potential of −0.4 V (vs. Ag/AgCl) in the electrolyte (0.5 M CuSO_4_ 5H_2_O) for 30 min (deposition temperature: 25 °C). Further electrodeposition of NiFe was performed to produce the NiFe-LDH electrocatalyst on the pyramids with the deposited Cu layer at an applied potential of − 1.3 V (vs. saturated calomel electrode, SCE) in the electrolyte (3 mM Ni(NO_3_)_2_ 6H_2_O and 3 mM Fe(NO_3_)_3_ 9H_2_O) for 15 min (deposition temperature: 10 °C). The deposition of Cu and NiFe was confirmed by the changing color of the 3D electrode shown in the optical images in Fig. 1.

### Effect of GMF concentration on rheological properties, printability, and conductivity

**Figure 2:**
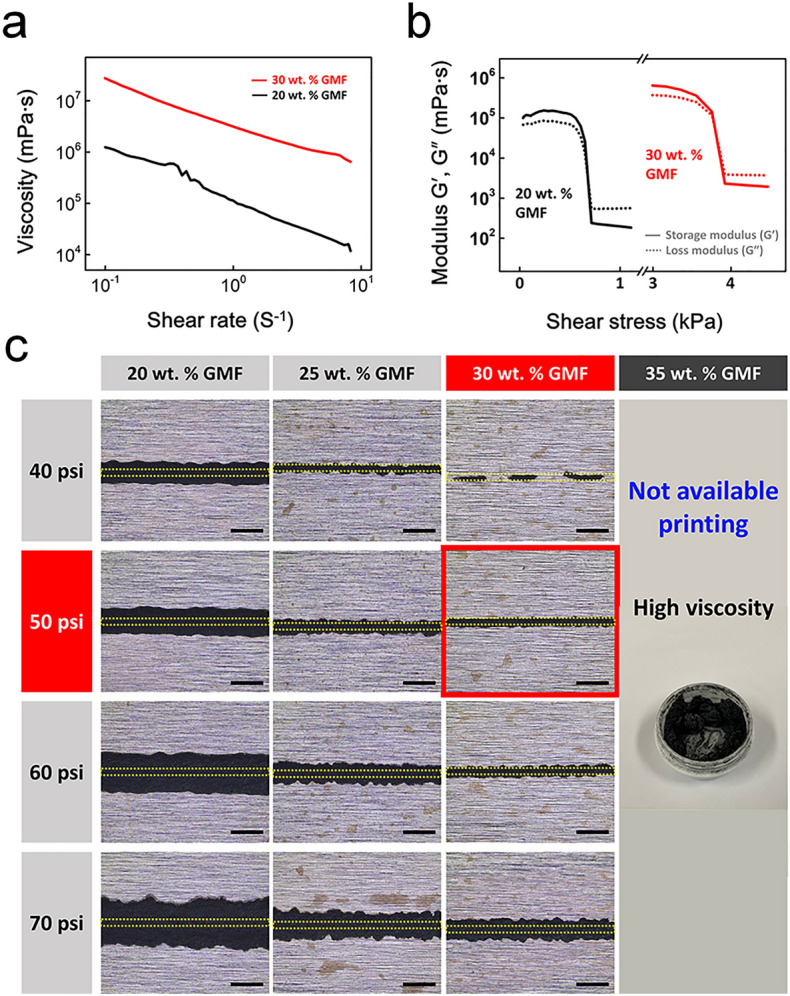
Rheological properties and printability of graphene inks. **(a)** Viscosity vs. shear rate. **(b)** Storage (G′) and loss (G˝) moduli *vs.* ink shear stress. **(c)** Ink printability as a function of GMF concentrations. Scale bar: 500 μm.

Printable graphene ink was studied for use with the ink-based 3D printing method, which requires ink with specific viscoelastic and rheological properties to facilitate extrusion through a nozzle under *P*. The GMFs with an average size of 7 μm in the ink conducted and supported the components, and ethyl cellulose (EC) acted as a filler to bind the GMFs. The viscosity and moduli of the graphene ink as a function of GMF concentration are shown in Fig. 2a,b. As the GMF concentration increased, the ink viscosity increased due to the agglomeration of GMFs. The inks with 20 and 30 wt.% GMF had viscosity values of 1.12 × 10^5^ and 3.14 × 10^6^ mPa s (at 1 s^−1^ shear rate) and showed a shear-thinning behavior (Fig. 2a). Figure 2b shows the storage (*G'*) and loss (*G’*) moduli of both inks. They exhibit rheological properties with transitions (shear yield stress) between solid-like (*G'* > *G''*) and fluid-like (*G'* < *G''*) behaviors with respect to the applied shear stress. As the graphene concentration increased, the yield stress also increased from 714 Pa (20 wt.% GMF) to 3923 Pa (30 wt.% GMF). Increased stiffness and yield stress of the inks with increasing graphene concentrations enables the printing of 3D microstructures but requires an increase in the *P* value during the process. *P* values should be optimized according to the graphene concentrations to obtain uniform structures.

Figure 2c shows the printability of the ink at GMF concentrations of 20, 25, 30, and 35 wt. % (Movie S2). The yellow dotted line in the optical images indicates the *ID* (100 μm) of the used nozzle. As the applied pressure increases, the printed line width (*W*_*L*_) also increases owing to the over-extrusion of the ink through the nozzle. At 20 wt.% GMF, as *P* increased from 40 to 70 psi, *W*_*L*_ increased from 340 to 760 μm, resulting in an uneven pattern. A uniform pattern with *W*_*L*_ similar to the inner diameter of the nozzle was produced at a printing condition of 30 wt.% GMF and 50 psi (indicated by the red square). At > 35 wt. % GMF, strong flake agglomeration induced nozzle clogging at all *P* ranges.

**Figure 3:**
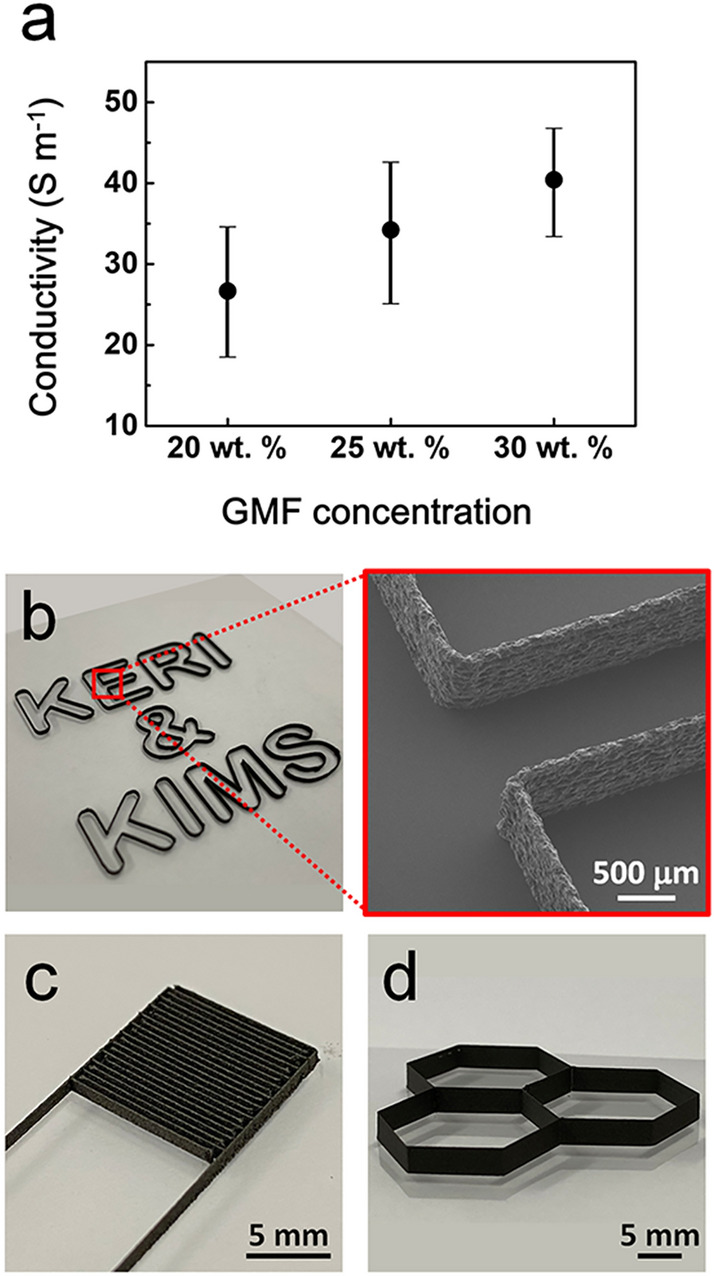
Conductivity of graphene inks and photographs of graphene 3D microstructures. **(a)** Ink conductivity as functions of graphene concentration. **(b–d)** Optical images of graphene 3D microstructures printed using 30 wt.% GMF. **(b)** KERI & KIMS, **(c)** 3D interdigitated, and **(d)** honeycomb architecture. Inset of **(b)** is a FE-SEM image of the 3D-printed “E” character.

We also investigated the change in the conductivity of the printed graphene line patterns as a function of GMF concentration (Fig. 3a). Graphene lines with a length of 120 mm were printed on a polyimide (PI) substrate. The conductivity of the printed lines at each concentration level was calculated as the average value of 10 patterns. Increasing GMF concentrations improved the conductivity of the pattern. At 30 wt.% GMF, the printed line exhibited an electrical conductivity of 41 S m^-1^. Graphene 3D microstructures with different shapes were fabricated on a polyethylene terephthalate substrate via layer-by-layer (LbL) 3D printing with the graphene ink. For LbL printing, the nozzle was alternately moved rightward and leftward, with the precise vertical movement (75 μm) of the nozzle. Figure 3b presents a structure corresponding to the letters of “KERI & KIMS,” consisting of flat and round walls with *W*_*L*_ of 100 μm and height of 600 μm. Enlarged SEM image of the “E” structure, indicated by the red square, shows a uniform and straight feature. 3D interdigitated (Fig. 3c; *W*_*L*_ = 100 μm, *h* = 1 mm, interspacing = 500 μm) and honeycomb (Fig. 3d; *W*_*L*_ = 100 μm, *h* = 3.3 mm) architectures were also successfully fabricated.

### Characterization of the 3D-printed pyramid electrode

**Figure 4:**
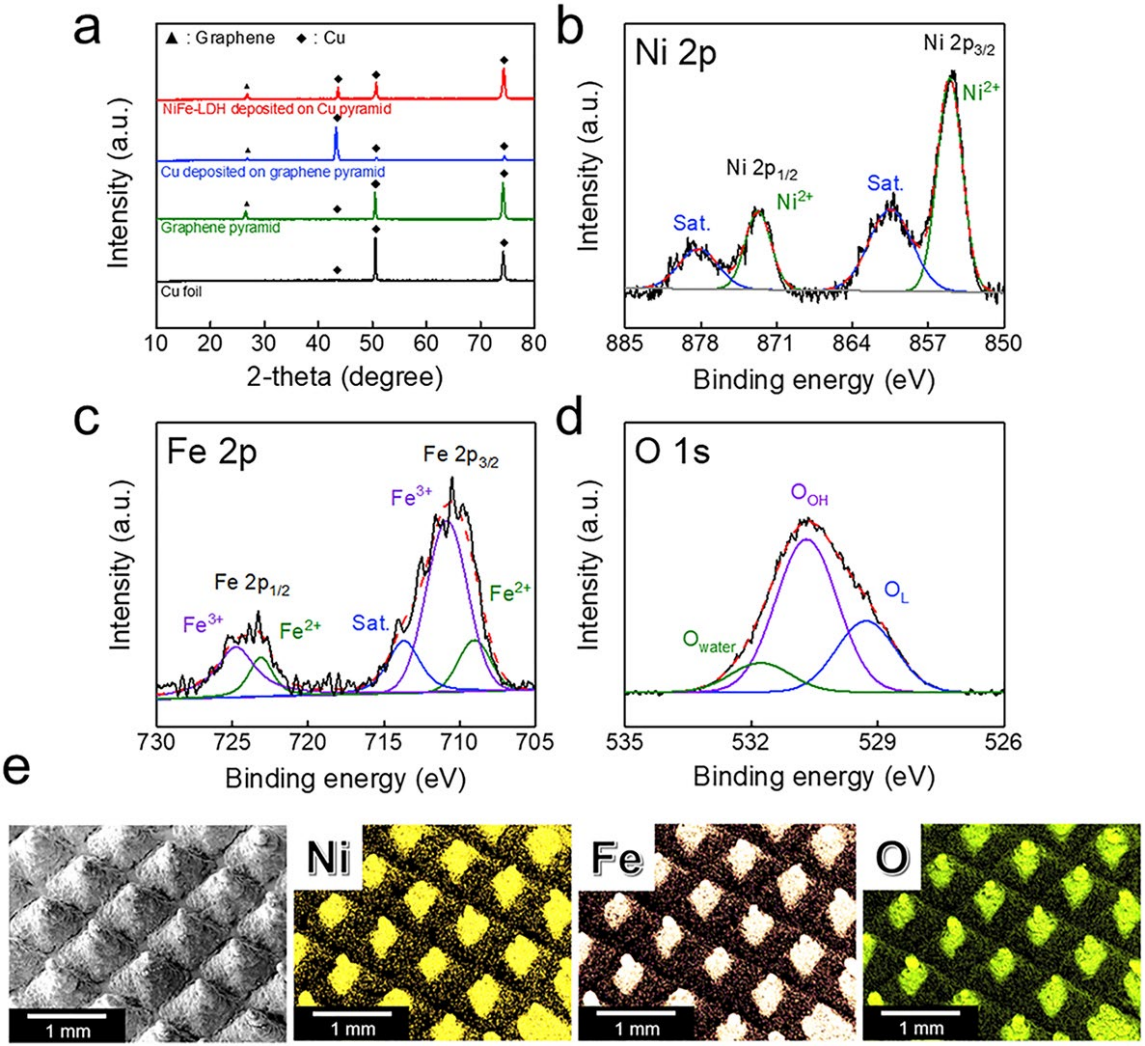
Physicochemical analysis of the fabricated 3D NiFe-LDH pyramid electrode. **(a)** X-ray diffraction pattern of the fabricated electrode: Cu-foil (black), graphene pyramid (green), Cu deposited on graphene pyramid (blue) and NiFe-LDH deposited on Cu pyramid (red). X-ray photoelectron spectra of **(b)** Ni 2p, **(c)** Fe 2p, and **(d)** O 1 s. **(e)** Energy dispersive spectrometry (EDS) mapping images of NiFe-LDH pyramid electrode: Ni (yellow), Fe (apricot), and O (green).

To investigate the physicochemical properties of the fabricated 3D pyramid electrode, the electrode was analyzed by X-ray diffraction (XRD), X-ray photoelectron spectroscopy (XPS), and energy dispersive spectrometry (EDS). In Fig. 4a, the XRD peaks of the Cu foil substrate show peaks at 43.4°, 50.5°, and 74.2° attributed to the (111), (002), and (022) planes (JCPDS No.98-004-3493), respectively. After printing the graphene pyramid array on the substrate, we could clearly observe an additional peak at 26.8°, which indicates graphene^31^. The pyramids with the electrodeposited Cu layer showed almost the same diffraction peak position as that of the as-printed graphene pyramid array, but the peak intensity exhibited different values due to the different dominant crystal orientations of the Cu foil (002) and electrodeposited Cu layer (111). This result is attributed to the preferential Cu growth in the (111) plane during electrodeposition^32^. After NiFe electrodeposition on the deposited Cu pyramids, no additional peaks were observed, indicating that the electrodeposited NiFe was amorphous in nature^33,34^. In order to confirm phase of LDH, we analyzed the transmission electron microscopy (TEM) images with selected area electron diffraction (SAED) pattern, as shown in Fig. S2. Since NiFe-LDH was deposited directly on the Cu layer, it was difficult to separate NiFe-LDH alone. Therefore, the phase of deposited NiFe-LDH was analyzed by obtaining a SAED pattern in the region where Cu was not present. The SAED pattern shows the (006) and (015) plane, which indicated NiFe is LDH structure.

XPS analysis was also performed to confirm NiFe deposition on the pyramids. The corresponding survey spectra are presented in Fig. S3. Figure 4b–d indicate the high-resolution spectra of Ni 2p, Fe 2p, and O 1 s, which means that Ni, Fe, and O are deposited on the surface of the pyramids^35–37^. The Ni 2p spectrum shows two spin–orbit peaks at 855 eV (Ni 2p_3/2_) and 872.8 eV (Ni 2p_1/2_) with satellite peaks, indicating the Ni^2+^ oxidation state. Fe 2p was deconvoluted into two peaks at 710.5 eV and 723.9 eV, corresponding to Fe 2p_3/2_ and Fe 2p_1/2_, respectively. In addition, Fe showed two oxidation states (Fe^2+^ and Fe^3+^); Fe^3+^ is a chemical state that plays an important role in forming LDH^38,39^. In O 1 s, oxygen was observed in three forms. The major peak O_OH_ is attributed to the M-OH bonding energy, which indicates hydroxyl species, and peak O_L_ corresponds to the lattice oxygen. A small peak of O_water_ is attributed to the physically adsorbed surface or residual water molecules^40,41^. The XPS analysis results showed that the electrodeposited NiFe has an LDH structure^33,42,43^. NiFe-LDHs have a superior activity that is comparable to the best noble catalysts for the OER^44,45^.

Figure 4e shows the EDS mapping results of the 3D pyramid electrode with the NiFe-LDH electrocatalyst for regular element distribution (Fig. S4 for magnified image). The electrodeposited NiFe formed on the pyramids is composed of nanoparticles with a diameter of approximately 35 ± 15 nm (Fig. S5). The elements Ni, Fe, and O are well distributed throughout the pyramid array. This, together with the 3D pyramid design of the electrode, can contribute to increasing the surface area of the catalyst.

### Electrochemical analysis of the 3D-printed pyramid electrode for OER

**Figure 5:**
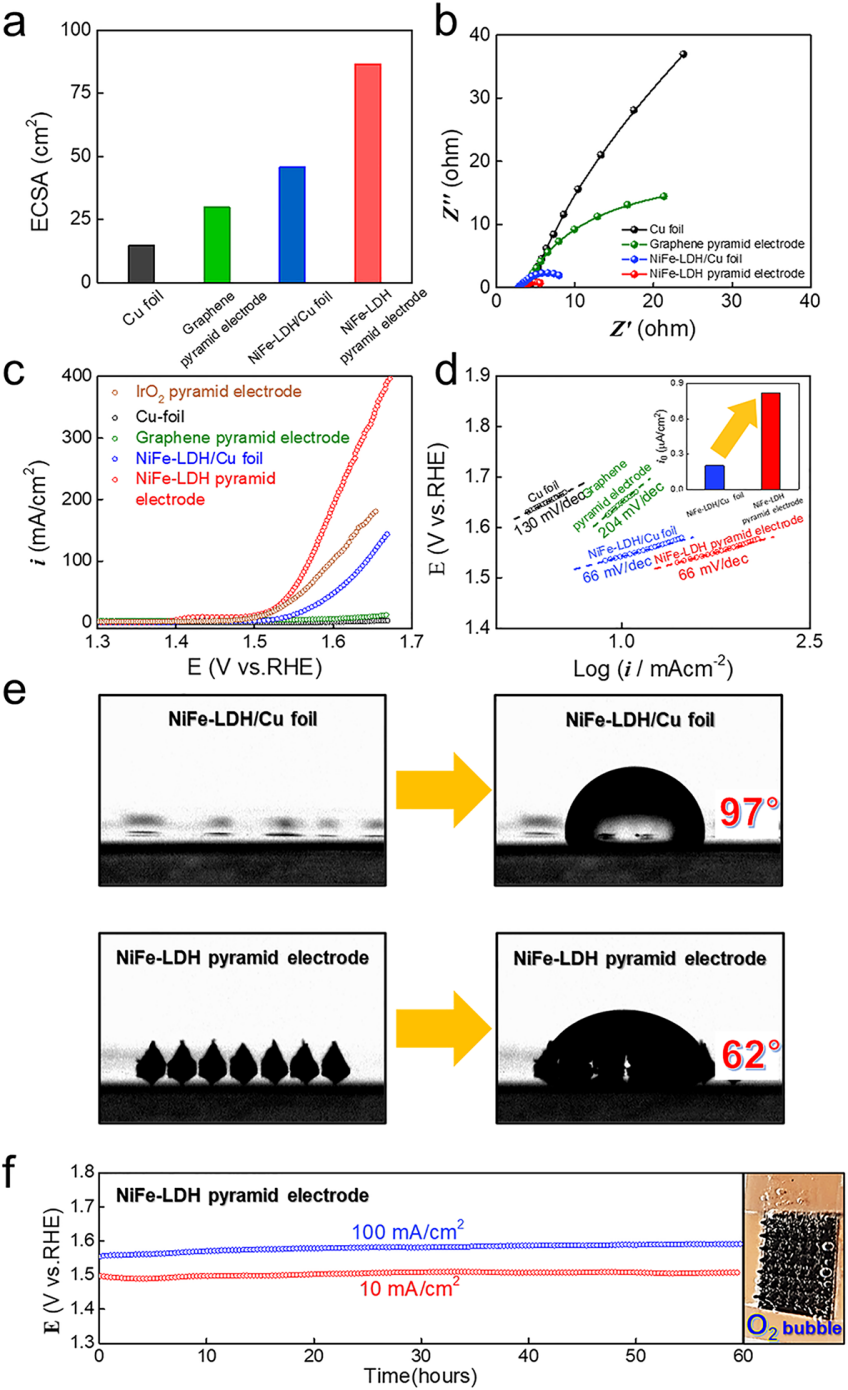
Electrochemical analysis for OER. **(a)** ECSA calculated from double layer capacitance. **(b)** Electrochemical impedance spectroscopy of Cu foil (black), graphene pyramid electrode (green), NiFe-LDH/Cu foil (blue) and NiFe-LDH pyramid electrode (red) at 1.58 V_RHE_. **(c)** 85% iR-corrected polarization curves of IrO_2_ on pyramid electrode (brown), Cu foil (black), graphene pyramid electrode (green), NiFe-LDH/Cu foil (blue) and NiFe-LDH pyramid electrode (red) in 1 M KOH. **(d)** Tafel plots obtained from iR-corrected polarization curves with exchange current density (inset). **(e)** Wettability of NiFe-LDH pyramid electrode and NiFe-LDH/Cu foil. **(f)** Durability test of NiFe-LDH pyramid electrode at 10 (red) and 100 (blue) mA/cm^2^ for 60 h with 85% iR correction.

We evaluated the electrocatalytic activity of the 3D-printed NiFe-LDH pyramid electrode by comparing the electrochemical behavior of the NiFe-LDH pyramid electrode with that of three types of electrodes with same base area of 0.6 × 0.6 cm^2^: the Cu foil, the graphene pyramid array, and the NiFe-LDH deposited Cu foil (NiFe-LDH/Cu foil). To compare the electrochemical surface area (ECSA) of each electrode, the double-layer capacitance (*C*_*dl*_) was calculated via cyclic voltammetry (CV) in the non-Faradaic region (Fig. S6). In Fig. 5a, the ECSA of the NiFe-LDH pyramid electrode exhibits the largest value among four types of electrodes, which is approximately twice as large as that of the NiFe-LDH/Cu foil. The electrochemical impedance spectroscopy (EIS) results indicate that the NiFe-LDH pyramid electrode significantly contributes to reducing the charge transfer resistance (*R*_*ct*_) in terms of the radius of the semicircle during the OER at 1.58 V (vs. RHE) (Fig. 5b). As shown in Fig. 5c, linear sweep voltammetry (LSV) was measured in 1 M KOH to evaluate the electrocatalytic activity of the synthesized catalysts. The Cu foil and graphene pyramid electrodes without the NiFe-LDH layer showed very poor OER activity. However, NiFe electrodeposition led to a dramatic improvement in their activity. The *η* value (258 mV at 10 mA/cm^2^) of the NiFe-LDH pyramid electrode was lower than that of the NiFe-LDH/Cu foil (314 mV at 10 mA/cm^2^). Although the OER onset potential was similar, as the electrode surface area provided by 3D pyramids increased, the active site of the electrode also increased, improving the OER activity. However, the Tafel slope of both the NiFe-LDH pyramid electrode and the NiFe-LDH/Cu foil was 66 mV/dec (Fig. 5d). The Tafel slope is significantly affected by the materials of the substrate because the electrocatalyst and the substrate materials interact strongly with each other. Therefore, observing the same Tafel slopes is reasonable because NiFe was electrodeposited on the Cu foil. In order to confirm the activity of the electrode, the exchange current density (*i*_*0*_) was obtained from the Tafel slope (inset of Fig. 5d). The *i*_*0*_ value (0.818 μA/cm^2^) of the NiFe-LDH pyramid electrode was four times larger than 0.203 μA/cm^2^ of the NiFe-LDH/Cu foil. This means that the increase in the electrode surface area also played an important role in improving the OER kinetics^46^.

Figure 5e shows the wettability of the NiFe-LDH pyramid electrode and the NiFe-LDH/Cu foil. In general, the hydrophilicity of the electrode surface is very advantageous for improving the mass transport in water splitting^11,12,47^. The contact angle of 62° for the NiFe-LDH pyramids was lower than 97° for the NiFe-LDH/Cu foil, indicating the hydrophilicity enhancement of the electrode due to geometrical effect. Thus, the NiFe-LDH pyramid electrode is efficient for mass transport in water splitting, showing higher activity than the NiFe-LDH/Cu foil. Among the factors of the electrocatalytic performance, durability is as important as activity. The durability of the OER in a chronopotentiometric response was confirmed in a 1 M KOH electrolyte. Based on the applied low and high current density process for the OER, the NiFe-LDH pyramid electrode showed excellent durability without potential decay for 60 h (Fig. 5f). After the long-term durability test (at -10 mA/cm^2^ for 300 h), the physical stability of the pyramid structure and the chemical states of the electrocatalyst were analyzed, as shown in Figures S7 and S8. Despite 300 h of durability testing, the microstructure of NiFe-LDH pyramid electrode was well maintained. This is a result showing that the electrode fabricated using 3D printing has good physical strength so that it can be used as water splitting electrode. In addition, we analyzed the chemical states of NiFe-LDH after durability tests (Fig. S8 and Table S1). The XPS peak position of Ni 2p and Fe 2p showed slightly positive change after durability test, which is due to the phase transformation of the catalyst surface into oxyhydroxide in a strong oxidizing environment^48,49^.

## Discussion

We experimentally demonstrated a 3D-printed NiFe-LDH pyramid electrode with higher OER performance than a flat electrode. The fabrication process of the 3D electrode is a three-step method, which consists of direct-ink-writing of a graphene pyramid array and successive electrodeposition of the Cu conductive layer and the NiFe-LDH electrocatalyst layer on the printed graphene pyramids. Graphene 3D pyramids (bottom area of 500 × 500 μm^2^, *h* of 600 μm, and inclination angle of 67.4°) were printed onto a flat square Cu foil (0.6 × 0.6 cm^2^) via extrusion-based 3D printing using a graphene ink (30 wt.% GMFs and the 70 wt.% polymer solution). The ink was stable and suitable for the 3D printing approach at a specific applied pressure (*P*). For efficient electrodeposition of the NiFe-LDH electrocatalyst on the 3D electrodes, Cu was deposited as a conductive layer onto the printed graphene pyramids. The electrodeposited NiFe was composed of nanoparticles with a diameter of ~ 35 ± 15 nm.

The proposed 3D pyramid electrode with a nanoparticle-based NiFe-LDH electrocatalyst had an ECSA twice as large as the flat electrode (NiFe-LDH/Cu foil). The *η* value of the NiFe-LDH pyramid electrode was 258 mV at 10 mA/cm^2^, which was lower than that of the NiFe-LDH/Cu foil (*η* = 314 mV at 10 mA/cm^2^). The *i*_*0*_ value (0.818 μA/cm^2^) of the pyramid electrode was also four times higher than the 0.203 μA/cm^2^ of the foil electrode. These results indicate that increasing the electrode surface area contributed to increasing the number of electrocatalytic active sites for the OER reaction, resulting in an improvement in the OER activity. In addition, our NiFe-LDH pyramid electrode was comparable to the previously reported NiFe LDH-based OER electrocatalyst (Table S2). The 3D-printed NiFe-LDH electrode also exhibited excellent durability without potential decay at both 10 and 100 mA/cm^2^ for 60 h. This 3D printing-based method is an effective approach for fabricating 3D electrodes that exhibit high electrocatalytic performance for water splitting applications.

## Methods

### Preparation of graphene ink

The graphene inks were prepared by mixing graphene microflakes (average size = 7 μm, Graphene Supermarket) with a polymer solution using a mixer (ARE-310, THINKY Corporation). The polymer solution is composed of ethyl cellulose (Sigma-Aldrich, assay 47.5–49.5%), toluene (Sigma-Aldrich, ≤ 99.9%), and xylene (Sigma-Aldrich, ≤ 98.5%) at a ratio of 4:13:13. The rheological properties of the inks were characterized using a rheometer (MCR102, Anton Paar) with a parallel-plate geometry configuration. A strain sweep experiment was conducted from 10^–1^ to 10 s^−1^ in order to measure the ink viscosity at varying shear rates, and a stress sweep experiment was conducted at a constant angular velocity of 10 rad/s to record the variations in the storage (G′) and loss (G′′) moduli of the ink as functions of sweep stress.

### 3D printing of graphene structures

The graphene structures were constructed using a fluid dispenser (Ultimus V, EFD Inc.) with a high-pressure dispensing tool (HPx, EFD). The graphene ink was packed into a syringe (3 mL barrel, EFD Inc.) and extruded through a micronozzle (nozzle inner diameter, *ID* = 100 μm) under an applied pressure (*P*). To optimize the printing conditions, the *P* value was tuned with respect to the concentration of graphene microflakes (GMFs) in the ink. The micronozzle position was accurately controlled using three-axis stepping motors, and the nozzle motion corresponding to the printed paths was designed using parameterized G-code scripts converted from the designed 3D model. The printing speed (υ) was maintained at 10 mm min^-1^ during the process. The printing process was observed in situ using an optical monitoring system consisting of an optical lens (10 ×) and a charge-coupled device camera. Cu foil (thickness: 20 μm), polyimide (PI, thickness: 200 μm), and polyethylene terephthalate (thickness: 200 μm) were used as substrates. The electrical conductivity of the printed graphene structures was measured using a two-probe method with a Keithley 2612A instrument.

### Electrodeposition of Cu and NiFe-LDH layers

For the electrodeposition of Cu and NiFe layers, a Pt mesh (3 cm × 4 cm) was used as a counter electrode. Cu electrodeposition was performed in an electrolyte of 0.5 M CuSO_4_ · 5H_2_O at a potential of -0.4 V (vs. Ag/AgCl (saturated KCl)) for 30 min at 25 °C. The deposited Cu electrode was rinsed with deionized (DI) water before drying in a convection oven at 70 °C. The NiFe layer was deposited onto the deposited Cu layer of the pyramids and the Cu foil (thickness: 20 μm) at an applied potential of − 1.3 V (*vs*. SCE, saturated calomel electrode) in the electrolyte (3 mM Ni(NO_3_)_2_ 6H_2_O and 3 mM Fe(NO_3_)_3_ 9H_2_O) for 15 min at 10 °C. The obtained NiFe electrode was rinsed with DI water before drying in a convection oven at 70 °C. For comparison of electrocatalytic activity, IrO_2_ was synthesized on the pyramid array by electrodeposition^50^.

### Characterization

The surface morphology of the samples was observed via field-emission scanning electron microscopy (FE-SEM, CZ/MIRAI LMH, TESCAN). XRD patterns (2θ = 10°–80°) were recorded at room temperature at a scanning speed of 1° min^-1^ using Cu Kα radiation (λ = 0.154 nm) (D/MAS-2500, Rigaku). X-ray photoelectron spectroscopy (XPS) was performed using ECSA2000 (VG Microtech.) on a dual Mg/Al X-ray source. Wettability was characterized using a contact measurement machine (NPT103, EPSM). All electrochemical measurements were conducted using an electrochemical workstation (VMP-3, Bio-Logic) in a three-electrode cell with 1 M KOH as the electrolyte at 25 °C. A Pt mesh (3 cm × 4 cm) and Hg/HgO (1 M KOH) were used as the counter and reference electrodes, respectively. All potentials were reported versus a reversible hydrogen electrode (RHE) using the Nernst equation. The OER activity was investigated via linear sweep voltammetry (LSV) at a scan rate of 5 mV/s. The LSV and durability results were 85% iR-corrected, and Tafel plots were obtained from those 85% iR-corrected LSV results. The electrochemical surface area (ECSA) was calculated from the double-layer capacitance (C_dl_) value using cyclic voltammetry (CV) at different scan rates in the non-Faradaic region in 1 M KOH (Eq. 1)^51^.

1 $${\text{ECSA }} = {\text{ C}}_{{{\text{dl}}}} /{\text{C}}_{{\text{s}}} ,$$

where C_s_ is the smooth plane capacitance for a metal surface, with a value of 40 µF/cm^2^. Electrochemical impedance spectroscopy (EIS) analysis was performed in 1 M KOH over the frequency range of 200 kHz to 10 Hz, with an amplitude of 10 mV at 1.58 V (vs. RHE). Durability tests were conducted at 10 and 100 mA/cm^2^ for 60 h in 1 M KOH.

## Supplementary Information

Supplementary Video 1.

Supplementary Video 2.

Supplementary Information.
