# Supplementary material for: 3D-printed NiFe-layered double hydroxide pyramid electrodes for enhanced electrocatalytic oxygen evolution reaction
Source: Sci Rep. 2022 Jan 10;12:346. doi: 10.1038/s41598-021-04347-9 (PMC8748972; doi:10.1038/s41598-021-04347-9)
Supplement: Supplementary file 3 — Supplementary Information. [file 41598_2021_4347_MOESM3_ESM.pdf]

Supplementary information

3D-Printed NiFe-Layered Double Hydroxide Pyramid Electrodes for Enhanced Electrocatalytic Oxygen Evolution Reaction

Jinhyuck Ahn[1,2,#], *Yoo Sei Park*[3,4,#], *Sanghyeon Lee*[5], *Juchan Yang*[3], *Jaeyeon Pyo*[1], *Jooyoung Lee*[3], *Geul Han Kim*[3,4], *Sung Mook Choi*[3,*] *and Seung Kwon Seol*[1,2,*]

[1]Smart 3D Printing Research Team, Korea Electrotechnology Research Institute (KERI), Changwon-si, Gyeongsangnam-do, 51543, Republic of Korea

[2]Electro-functional Materials Engineering, University of Science and Technology (UST), Changwon-si, Gyeongsangnam-do, 51543, Republic of Korea

[3]Department of Energy & Electronic Materials, Surface Materials Division, Korea Institute of Materials Science, Changwon-si, Gyeongsangnam-do, 642831, Republic of Korea.

[4]Department of Materials Science and Engineering, Pusan National University, Busan, 46241, Republic of Korea.

[5]KIURI Institute, Yonsei University, Seoul, 03722, Republic of Korea

Corresponding Authors

*E-mail addresses: akyzaky@kims.re.kr (S. M. Choi) and skseol@keri.re.kr (S. K. Seol)

Author Contributions

[#]These authors contributed equally to this work.

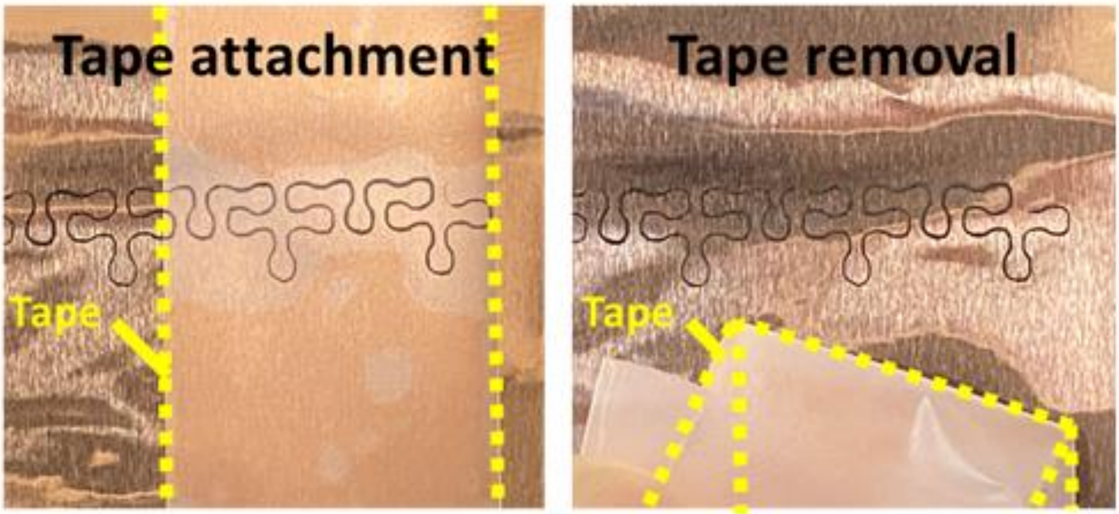

Figure S1. Tape test to verify the adhesion of the graphene pattern to Cu foil substrate. The printed graphene pattern provides good adhesion to the substrates.

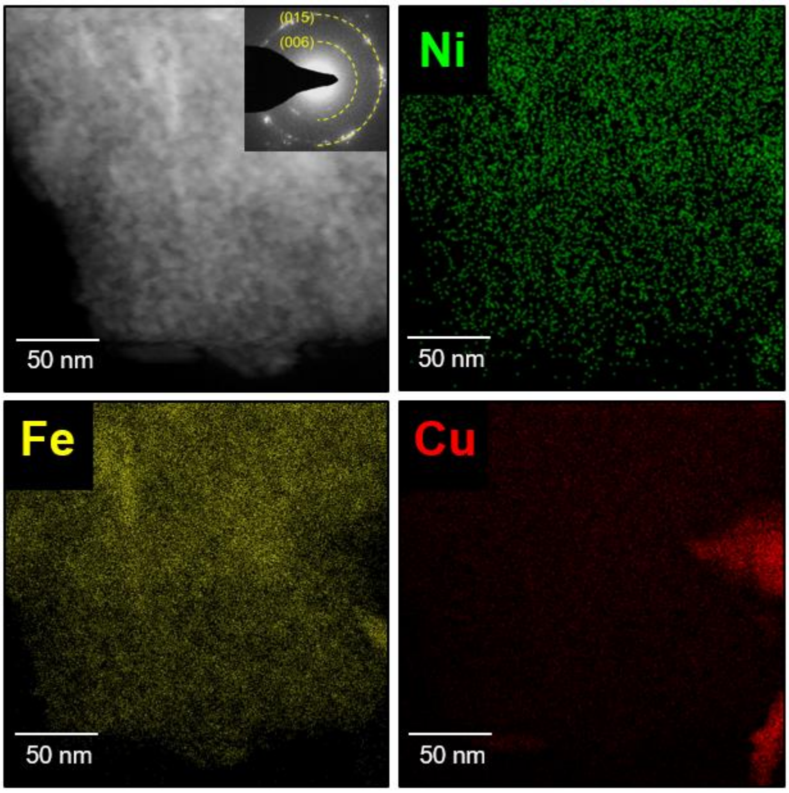

Figure S2. Transmission electron microscopy (TEM) image and the corresponding energy-dispersive X-ray spectroscopy (EDS) elemental mapping images of 3D-printed NiFe LDH pyramid electrode. Selected Area Electron Diffraction (SAED) pattern was obtained in the region where Cu was not observed.

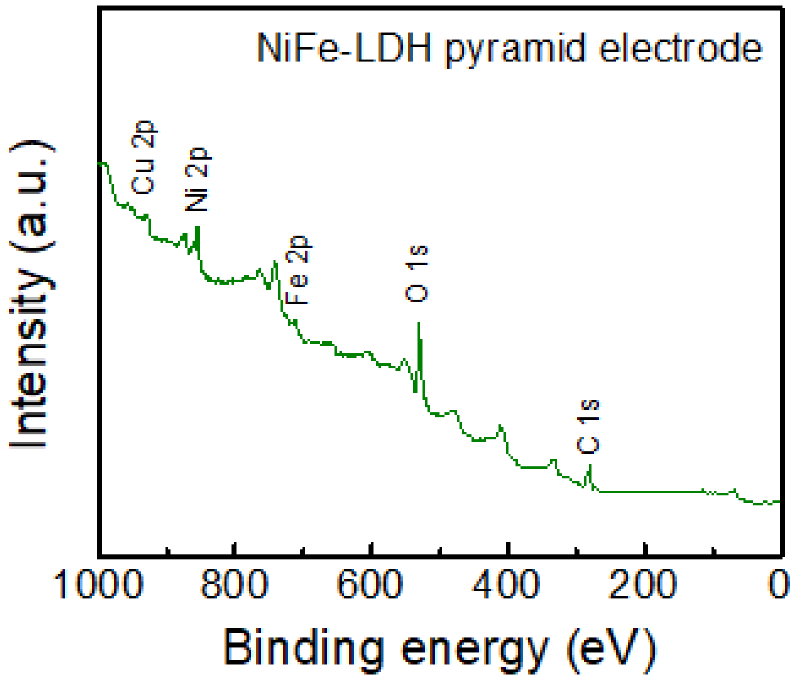

Figure S3. XPS survey spectrum of NiFe-LDH pyramid electrode.

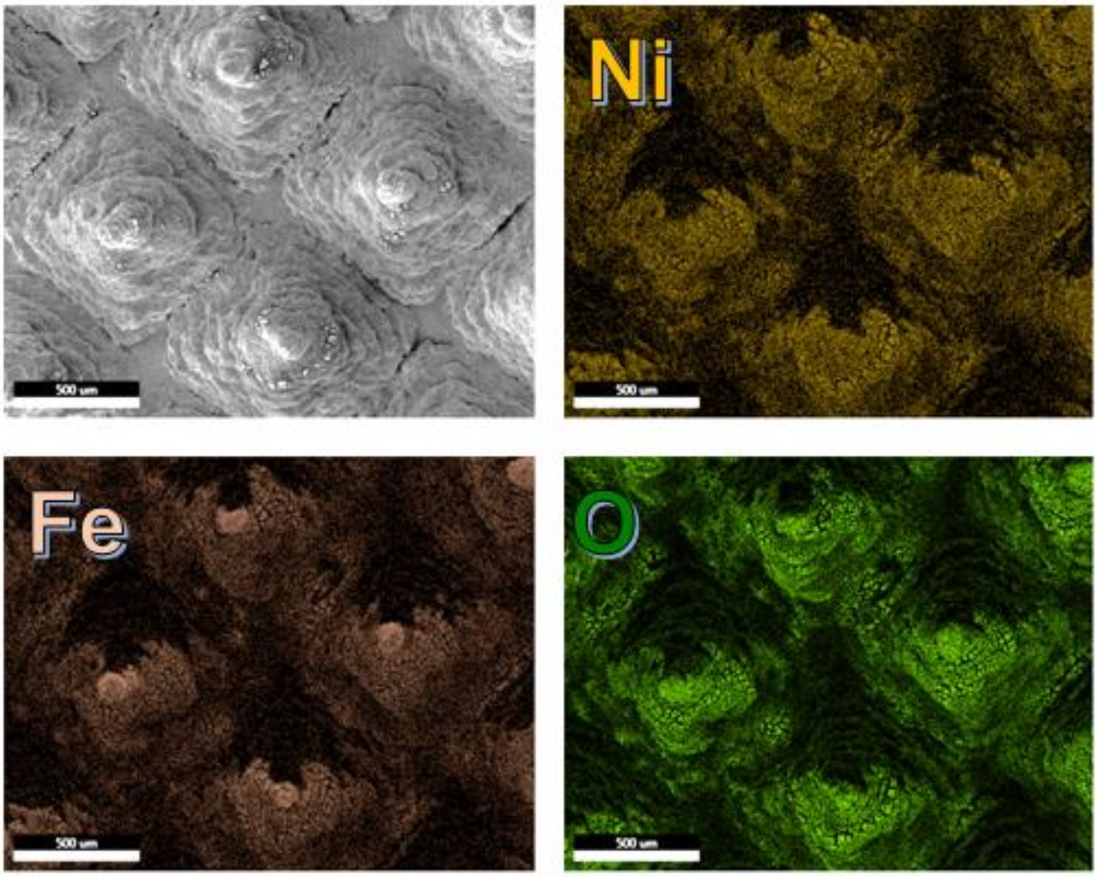

Figure S4. Energy dispersive spectrometry (EDS) mapping images of NiFe-LDH pyramid electrode: Ni (yellow), Fe (apricot), and O (green).

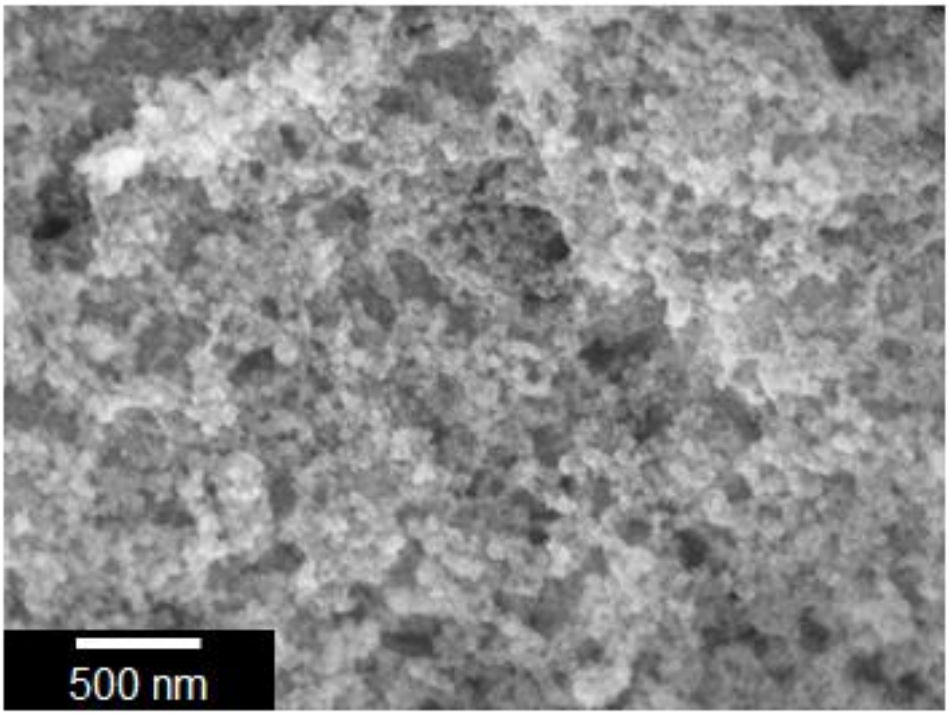

Figure S5. High-resolution FE-SEM image of NiFe-LDH pyramid electrode.

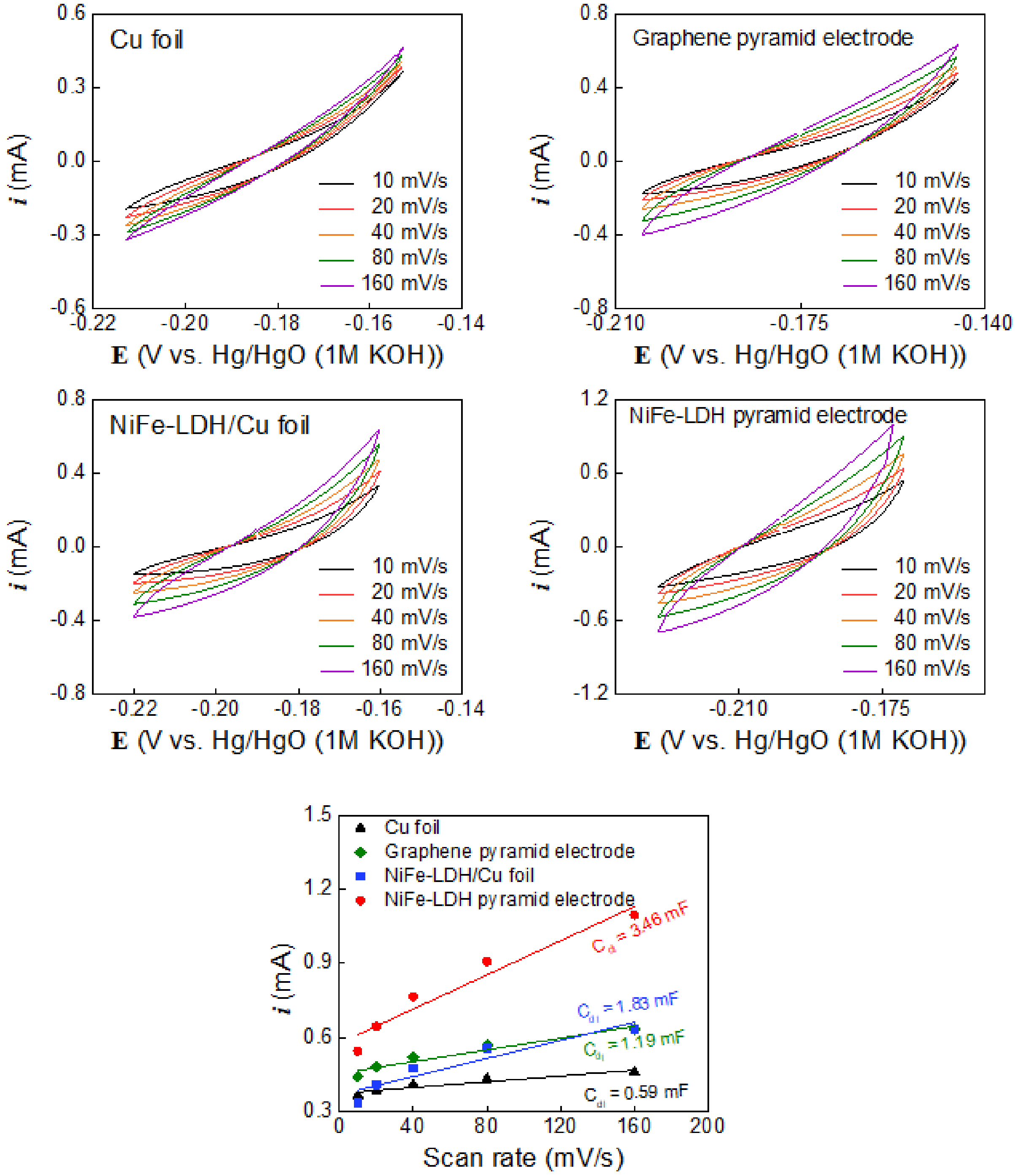

Figure S6. Double layer capacitance measurement of Cu foil, graphene pyramid electrode, NiFe-LDH/Cu foil and NiFe-LDH pyramid electrode in the non-Faradaic region in 1M KOH with different scan rates.

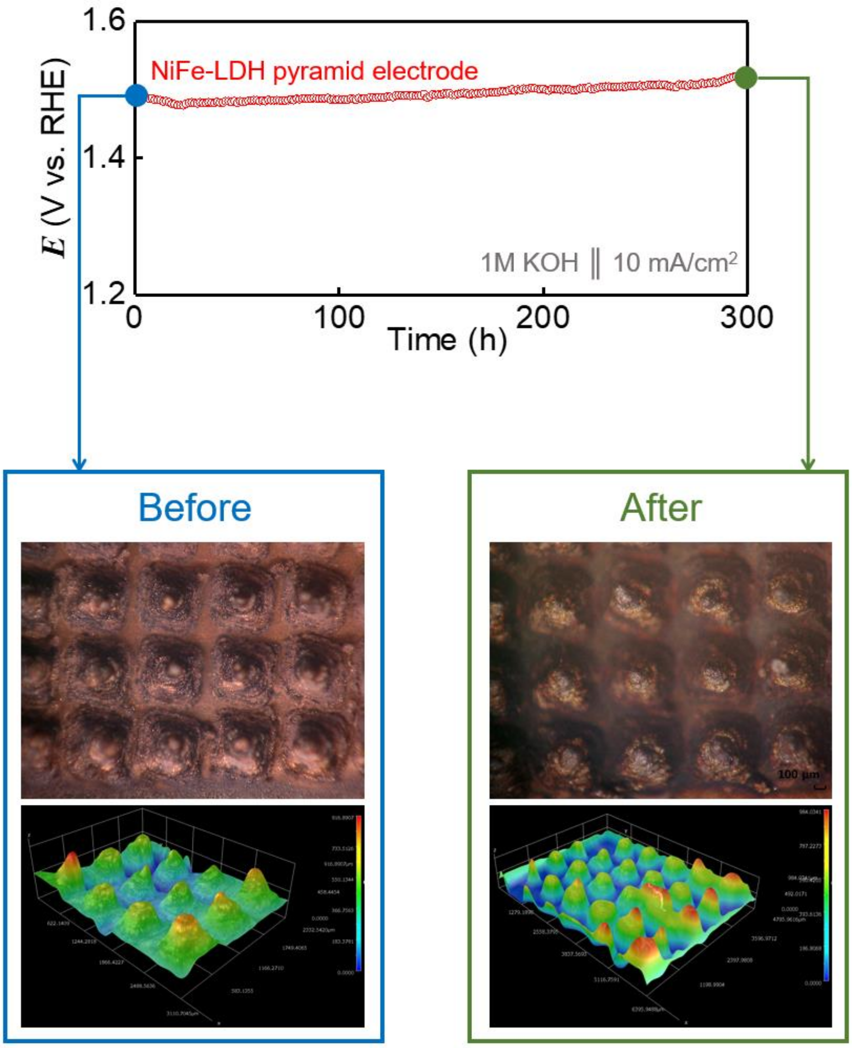

Figure S7. The durability test for NiFe-LDH pryamid electrode at constant current in 1 M KOH. The morphology change of the electrode after durability test was confirmed with an optical microscope. The upper image is a top view, and the below image is a 3D digital microscope result of the electrode. It was confirmed that the pyramid structure was maintained even after the durability test.

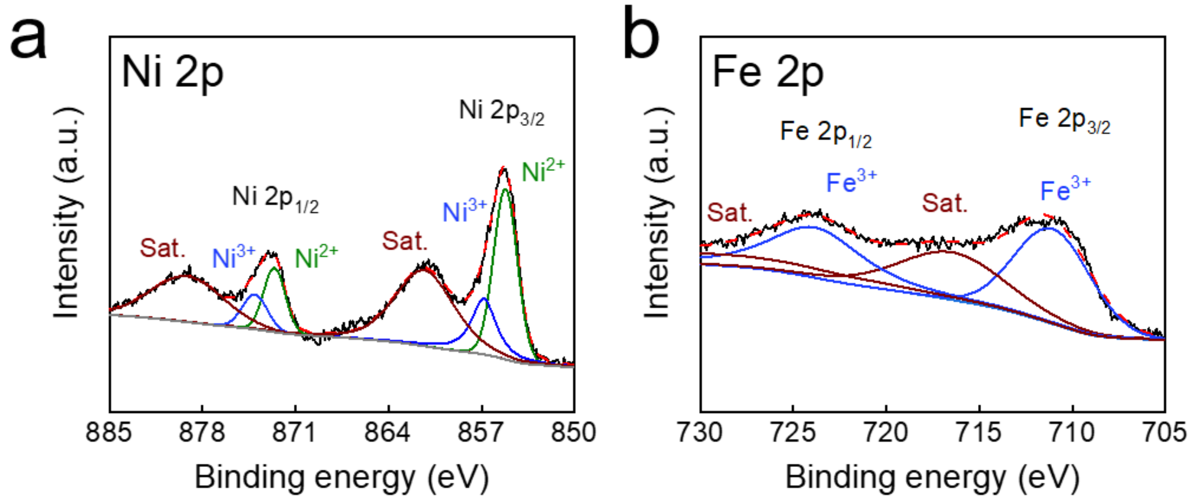

Figure S8. XPS analysis of NiFe on pyramid array after durability test: (a) Ni 2p and (b) Fe 2p.

Table S1. XPS binding energy of Ni 2p and Fe 2P before/after durability test.

	**Ni**		**Fe**	
	**Ni $2p_{3/2}$ (eV)**	**Ni $2p_{1/2}$ (eV)**	**Fe $2p_{3/2}$ (eV)**	**Fe $2p_{1/2}$ (eV)**
Before	855.0	872.8	710.5	723.9
After	855.3	872.9	711.6	724.1

Table S2. Comparison of the OER activity.

OER electroctalyst	Overpotential [@ 10 mA/cm^2]	Electrolyte	Reference
**NiFe-LDH on pyramid array**	**258 mV**	**1 M KOH**	**This work**
$NiFe^{0.15M}$ LDH	272 mV	1 M KOH	[1]
NiFeMn LDH	290 mV (@ 20 mA/cm^2)	1 M KOH	[2]
FeOOH on Fe-foil	274 mV	1 M KOH	[3]
NiFe LDH	269 mV	1 M KOH	[4]
NiCoFe-LDH	260 mV	1 M KOH	[5]
NiO/C@ NiFe-LDH	299 mV	1 M KOH	[6]
NiFe–$NiFe_2O_4$	316 mV	1 M KOH	[7]
NiFe@PCN	310 mV	1 M KOH	[8]
NiFe LDH	300 mV	1 M KOH	[9]
NiFe@NC	297 mV	1 M KOH	[10]
NiFe-carbon	296 mV	1 M KOH	[11]
NiFeCr-LDH	280 mV	1 M KOH	[12]
FeNi-LDH /Ti_3C_2-MXene	298 mV	1 M KOH	[13]
NiFe/NiFe : Pi	290 mV	1 M KOH	[14]
Monolayer NiFe LDH	254 mV	1 M KOH	[15]
NiFe-LDH	282 mV	1 M KOH	[16]

References

[1] S.H. Kim, Y.S. Park, C. Kim, I.Y. Kwon, J. Lee, H. Jin, Y.S. Lee, S.M. Choi, Y. Kim, Self-assembly of Ni–Fe layered double hydroxide at room temperature for oxygen evolution reaction, Energy Reports, 6 (2020) 248-254.

[2] Z. Lu, L. Qian, Y. Tian, Y. Li, X. Sun, X. Duan, Ternary NiFeMn layered double hydroxides as highly-efficient oxygen evolution catalysts, Chemical Communications, 52 (2016) 908-911.

[3] J. Zou, G. Peleckis, C.-Y. Lee, G.G. Wallace, Facile electrochemical synthesis of ultrathin iron oxyhydroxide nanosheets for the oxygen evolution reaction, Chemical Communications, 55 (2019) 8808-8811.

[4] Y. Liu, X. Liang, L. Gu, Y. Zhang, G.-D. Li, X. Zou, J.-S. Chen, Corrosion engineering towards efficient oxygen evolution electrodes with stable catalytic activity for over 6000 hours, Nature Communications, 9 (2018) 2609.

[5] L. Qian, Z. Lu, T. Xu, X. Wu, Y. Tian, Y. Li, Z. Huo, X. Sun, X. Duan, Trinary Layered Double Hydroxides as High-Performance Bifunctional Materials for Oxygen Electrocatalysis, Advanced Energy Materials, 5 (2015) 1500245.

[6] X. Li, M. Fan, D. Wei, X. Wang, Y. Wang, Core-Shell NiO/C@ NiFe-LDH Nanocomposite as an Efficient Electrocatalyst for Oxygen Evolution Reaction, Journal of the Electrochemical Society, 167 (2020) 024501.

[7] R.A. Raimundo, V.D. Silva, E.S. Medeiros, D.A. Macedo, T.A. Simões, U.U. Gomes, M.A. Morales, R.M. Gomes, Multifunctional solution blow spun NiFe–NiFe2O4 composite nanofibers: Structure, magnetic properties and OER activity, Journal of Physics and Chemistry of Solids, 139 (2020) 109325.

[8] C. Wu, X. Zhang, Z. Xia, M. Shu, H. Li, X. Xu, R. Si, A.I. Rykov, J. Wang, S. Yu, S. Wang, G. Sun, Insight into the role of Ni–Fe dual sites in the oxygen evolution reaction based on atomically metal-doped polymeric carbon nitride, Journal of Materials Chemistry A, 7 (2019) 14001-14010.

[9] L. Dang, H. Liang, J. Zhuo, B.K. Lamb, H. Sheng, Y. Yang, S. Jin, Direct Synthesis and Anion Exchange of Noncarbonate-Intercalated NiFe-Layered Double Hydroxides and the Influence on Electrocatalysis, Chemistry of Materials, 30 (2018) 4321-4330.

[10] Y. Zhang, X. Xia, X. Cao, B. Zhang, N.H. Tiep, H. He, S. Chen, Y. Huang, H.J. Fan, Ultrafine Metal Nanoparticles/N-Doped Porous Carbon Hybrids Coated on Carbon Fibers as Flexible and Binder-Free Water Splitting Catalysts, Advanced Energy Materials, 7 (2017) 1700220.

[11] R.A. Raimundo, V.D. Silva, T.R. Silva, E.S. Medeiros, D.A. Macedo, U.U. Gomes, R.M. Gomes, M.A. Morales, Synthesis and characterization of NiFe-carbon fibers by solution blow

spinning and application for the oxygen evolution reaction, Journal of Physics and Chemistry of Solids, 160 (2022) 110311.

[12] Y. Yang, L. Dang, M.J. Shearer, H. Sheng, W. Li, J. Chen, P. Xiao, Y. Zhang, R.J. Hamers, S. Jin, Highly Active Trimetallic NiFeCr Layered Double Hydroxide Electrocatalysts for Oxygen Evolution Reaction, Advanced Energy Materials, 8 (2018) 1703189.

[13] M. Yu, S. Zhou, Z. Wang, J. Zhao, J. Qiu, Boosting electrocatalytic oxygen evolution by synergistically coupling layered double hydroxide with MXene, Nano Energy, 44 (2018) 181-190.

[14] Y. Li, C. Zhao, Enhancing Water Oxidation Catalysis on a Synergistic Phosphorylated NiFe Hydroxide by Adjusting Catalyst Wettability, ACS Catalysis, 7 (2017) 2535-2541.

[15] Y. Zhao, X. Zhang, X. Jia, G.I.N. Waterhouse, R. Shi, X. Zhang, F. Zhan, Y. Tao, L.-Z. Wu, C.-H. Tung, D. O'Hare, T. Zhang, Sub-3 nm Ultrafine Monolayer Layered Double Hydroxide Nanosheets for Electrochemical Water Oxidation, Advanced Energy Materials, 8 (2018) 1703585.

[16] Y. Bi, Z. Cai, D. Zhou, Y. Tian, Q. Zhang, Q. Zhang, Y. Kuang, Y. Li, X. Sun, X. Duan, Understanding the incorporating effect of Co2+/Co3+ in NiFe-layered double hydroxide for electrocatalytic oxygen evolution reaction, Journal of Catalysis, 358 (2018) 100-107.

Movie S1: 3D printing of the graphene pyramid structures.

Movie S2: Printability of the ink at GMF concentrations of 20, 25, 30, and 35 wt. %.
